# Application of metagenomic next-generation sequencing in the etiological diagnosis of refractory pneumonia in children

**DOI:** 10.3389/fmicb.2024.1357372

**Published:** 2024-07-15

**Authors:** Ya-nan Wang, Yu-ting Wu, Ling Cao, Wen-quan Niu

**Affiliations:** ^1^Department of Respiratory Medicine, The Children's Hospital Affiliated to the Capital Institute of Paediatrics, Beijing, China; ^2^Department of Paediatrics, Luoyang Central Hospital Affiliated to Zhengzhou University, Luoyang, China; ^3^Center for Evidence-Based Medicine, Capital Institute of Paediatrics, Beijing, China

**Keywords:** metagenomic next-generation sequencing, child, refractory pneumonia, etiological diagnosis, pathogen

## Abstract

**Objective:**

Metagenomic next-generation sequencing (mNGS) was used to analyze the etiological distribution of refractory pneumonia in children. We compared its efficacy in pathogen diagnosis against traditional methods to provide a basis for clinical adjustment and treatment.

**Methods:**

A total of 60 children with refractory pneumonia treated at the Department of Respiratory Medicine, Children’s Hospital Affiliated with the Capital Institute of Paediatrics, from September 2019 to December 2021 were enrolled in this study. Clinical data (including sex, age, laboratory tests, complications, and discharge diagnosis) and lower respiratory tract specimens were collected, including bronchoalveolar lavage fluid (BALF), deep sputum, pleural effusion, lung abscess puncture fluid, traditional respiratory pathogens (culture, acid-fast staining, polymerase chain reaction, serological testing, etc.), and mNGS detection methods were used to determine the distribution of pathogens in children with refractory pneumonia and to compare the positive rate and diagnostic efficiency of mNGS and traditional pathogen detection for different types of pathogens.

**Results:**

Among the 60 children with refractory pneumonia, 43 specimens were positive by mNGS, and 67 strains of pathogens were detected, including 20.90% (14 strains) of which were *Mycoplasma pneumoniae*, 11.94% (8 strains) were *Streptococcus pneumoniae*, 7.46% (5 strains) were cytomegalovirus, and 5.97% (4 strains) were *Candida albicans*. Thirty-nine strains of *Mycoplasma pneumoniae* (41.03%, 16 strains), *Streptococcus pneumoniae* (10.26%, 4 strains), *Candida albicans* (7.69%, 3 strains), and Aspergillus (5.13%, 2 strains) were detected using traditional methods. The positive rate of mNGS detection was 90.48%, and the positive rate of the traditional method was 61.90% (*p* = 0.050), especially for G+ bacteria. The positive rate of mNGS was greater than that of traditional methods (*p* < 0.05), but they had no significant difference in detecting G- bacteria, viruses, fungi, or Mycoplasma/Chlamydia. Among the 60 patients, 21 had mixed infections, 25 had single infections, and the other 14 had unknown pathogens. *Mycoplasma pneumoniae* was most common in both mixed infections and single infections. The sensitivity, specificity, positive predictive value, and negative predictive value of mNGS were 95.45, 37.50, 80.77, and 75.00%, respectively. The sensitivity, specificity, positive predictive value, and negative predictive value of the traditional methods were 72.72, 62.50, 84.21, and 45.45%, respectively. The clinical compliance of mNGS was 80.00%, and that of the traditional method was 70.00%. The sensitivity and negative predictive value of mNGS were high, and the difference in the sensitivity for detecting G+ bacteria was statistically significant (*p* < 0.05). However, the differences in G- bacteria, fungi, and Mycoplasma/Chlamydia were not statistically significant (*p* > 0.05). Due to the small sample size, statistical analysis could not be conducted on viral infections.

**Conclusion:**

mNGS has higher overall efficacy than traditional methods for the etiological diagnosis of refractory pneumonia in children. The application of mNGS can significantly improve the detection rate of pathogens in children with refractory pneumonia. The sensitivity and negative predictive value of mNGS for detecting G+ bacteria are greater than those of other methods, and it can exclude the original suspected pathogenic bacteria. Unnecessary antibiotic use was reduced, but there was no statistically significant difference in G- bacteria, fungi, or Mycoplasma/Chlamydia.

## 1 Introduction

Pneumonia is a highly prevalent disease in children. According to the World Health Organization’s statistics on the causes of death in children under 5 years of age, 13.3% of children died of pneumonia in 2019, making it one of the leading causes of death in children under 5 years of age (GBD 2019 Under-5 Mortality Collaborators, 2021). With the development of medical care and improvements in social living standards, the diagnosis and treatment of pediatric pneumonia have significantly improved (He et al., 2017). Refractory pneumonia still has high morbidity and mortality in the clinic, which places a heavy burden on society and families (Smalley et al., 2012; Darby et al., 2017; Torres et al., 2019). Although technologies for pathogen detection are relatively mature, many refractory pneumonia pathogens still cannot be detected, which makes treatment difficult. Traditional detection methods mainly include morphological detection, culture separation, biochemical detection, immunology, and nucleic acid detection. These methods are simple to perform, have relatively low detection costs, have good sensitivity and specificity, and are thus still widely used in clinical practice. However, traditional detection methods are time-consuming, can only detect a wide variety of common infectious pathogens at once, and rely largely on the judgment of clinicians, so they cannot easily identify unknown or rare pathogens. Metagenomic next-generation sequencing (mNGS) technology is a DNA/RNA sequencing method developed in recent years. It has the advantages of high speed, high sensitivity, and no bias. In theory, it can detect all potential pathogens in samples and provide a new means for the clinical diagnosis of infectious diseases. Therefore, this study aimed to analyze the composition of pathogenic microorganisms in children with refractory pneumonia treated at the Department of Respiratory Medicine, Children’s Hospital Affiliated with the Capital Institute of Paediatrics, by using mNGS and to compare the differences in performance between mNGS and traditional respiratory pathogen detection methods to provide a reference for the clinical application of mNGS.

## 2 Methods

The data from children with refractory pneumonia who were hospitalized in the Department of Respiratory Medicine, Children’s Hospital Affiliated with the Capital Institute of Paediatrics, from September 2019 to December 2021 were collected. We collected etiological specimens from the lower respiratory tract and performed traditional laboratory tests (including culture, acid-fast staining, PCR, and serological detection) as well as mNGS. Clinical data, including sex, age, symptoms and signs, complications, inflammatory indices (white blood cell count, c-reactive protein, and calcitonin original), and discharge diagnosis, were also collected.

Children who met the diagnostic criteria for refractory pneumonia (Simner et al., 2018) were included in our study, and the diagnoses included severe pneumonia with poor response to treatment, prolonged pneumonia, and slow absorption pneumonia. The diagnostic criteria were as follows: (1) severe pneumonia refers to severe ventilation and/or gas exchange dysfunction or intrapulmonary and extrapulmonary complications (Burnham et al., 2014); (2) prolonged treatment refers to a > 2-week course of the disease, ineffective after active treatment, deterioration of the disease, and prolonged recovery; and (3) slow absorption refers to patients whose symptoms and signs improved after treatment, but reexamination of chest imaging after 2 weeks showed that the absorption of the lesions was less than 50%. Children with non-infectious diseases such as lung-targeting autoimmune diseases, pulmonary vascular diseases, pulmonary edema caused by heart failure, atelectasis caused by airway obstruction, bronchial foreign bodies, aspiration pneumonia, allergic lung disease, diffuse interstitial lung disease, eosinophilic pneumonia, or incomplete clinical data were excluded.

The collected specimens included alveolar lavage fluid, sputum, pleural effusion fluid, and lung abscess puncture fluid. (1) Collection of alveolar lavage solution: 2–5 mL of lavage solution was collected from the lesion site by electronic bronchoscopy, and alveolar lavage fluid was collected from all the children without artificial airways. (2) Sputum specimen collection: For children with artificial airways, a disposable sputum collector was used to aspirate sputum, and 3–5 mL was reserved. (3) Collection of puncture fluid for pleural effusion and lung abscess: 3–5 mL of puncture fluid for pleural effusion or lung abscess was collected through pleural puncture or closed thoracic drainage under strict aseptic operation. The collected specimens were divided into two samples: one was sent to our hospital for traditional pathogen detection, and the other was stored at −20°C and sent to a sequencing company for mNGS detection. At present, there is no unified standard for the interpretation of mNGS results under non-sterile respiratory conditions.

All the results were interpreted by clinicians based on the medical history, clinical characteristics, and auxiliary examination of the children. (1) The quality of the report and the credibility of the results were analyzed. It was determined whether the test results were standardized and credible according to the specimen type, report form, and content of the report. (2) The microorganisms detected by mNGS were classified as pathogenic microorganisms, conditional pathogenic microorganisms, or colonized microorganisms. Pathogenic microorganisms have a low potential to colonize the lung, and positive results are considered the causative agent of infection. Conditional pathogenic microorganisms should be further analyzed for host factors, blood physical and chemical indices, imaging characteristics, history of anti-infective drug use, treatment response, and traditional microbiological test results. The likelihood of respiratory tract infection is low, and respiratory tract infection is not considered pathogenic; however, when aspiration pneumonia, lung abscess, empyema, and other mixed infections occur, oral colonizing microorganisms may also cause disease. Skin-colonizing microorganisms detected in percutaneous lung biopsy specimens or pleural effusion specimens are usually not pathogenic. (3) Clinical decision of negative mNGS results: If the mNGS results are negative but the comprehensive clinical characteristics are still strongly suspected of being infectious, it is recommended to analyze the background microorganisms, raw data list, or other pathogenic microorganism test results in the report. If the clinical features support a non-infectious disease, the diagnosis and treatment of the primary disease should be made. (4) A multidisciplinary consultation (MDT) should be arranged: If the pathogen cannot be confirmed after the above process, an MDT discussion can be organized. When a variety of pathogenic microorganisms are detected by mNGS, confirmation by microscopic examination, culture, antigen detection, PCR, and other methods combined with the interpretation of clinical characteristics is recommended. If necessary, the results of the mNGS of different specimens can be mutually verified. The following clinical scenarios mostly support that multiple pathogenic microorganisms detected by mNGS have true positive results: (1) Patients with aspiration risk or a clear history of aspiration have a variety of common oral colonization bacteria, and aspiration pneumonia/lung abscess should be considered. Such cases can often be detected in lower respiratory tract specimen smears and/or cultures. (2) CAP patients sometimes develop mixed infections with bacteria, atypical pathogens, and respiratory viruses. (3) Mixed infections caused by bacteria, fungi, viruses, mycobacteria, and other pathogenic microorganisms often occur in the respiratory tract of immunocompromised hosts (Chinese Thoracic Society, 2023). However, the small sample size of our RNA sequencing data during the study period may have affected the results, so we only collected the DNA sequencing results in this study.

The pathogen detection steps were as follows: (1) fungal or bacterial culture: suspected fungal-infected patients were tested by sputum or bronchoalveolar lavage fluid (BALF) for fungal culture; sputum, pleural effusion, or BALF was collected and inoculated on Petri dishes (automatic culture instrument purchased from BD, USA, and sterile sputum culture bottles purchased from Oxoid, UK); and (2) antibody detection: a gelatine particle agglutination test was used to detect antibody titers (the kit was manufactured by Fujie Bio Inc., Japan). (3) Pathogen nucleic acid detection: Nucleic acid was extracted using the nucleic acid extraction QIAamp MinElute virus spin kit (Qiagen GmbH, Germany). The polymerase chain reaction (PCR) test was conducted using the NxTAG™ RPP kit purchased from Luminex, Canada, or the thermostatic amplification chip method using the kit purchased from Beijing Boao Biological Group Co., Ltd., Item No. 360090.

Samples were collected from patients according to standard procedures. DNA was extracted using the TIANamp Magnetic DNA Kit (Tiangen) according to the manufacturer’s protocols. The quantity and quality of the DNA were assessed using a Qubit (Thermo Fisher Scientific) and a NanoDrop (Thermo Fisher Scientific), respectively. DNA libraries were prepared using the KAPA Hyper Prep Kit (KAPA Biosystems) according to the manufacturer’s protocols. We used an in-house-developed bioinformatics pipeline for pathogen identification. Briefly, high-quality sequencing data were generated by removing low-quality reads, adapter contaminants, and duplicated and short reads (length < 36 bp). Human host sequences were identified by mapping to the human reference genome (hs37d5) using Bowtie2 software. Reads that could not be mapped to the human genome were retained and aligned with the microorganism genome database^1^ for pathogen identification.

The following etiological diagnostic criteria were used: (1) bacterial infection, in which the bacterial cultures or nucleic acid results were positive, and clinical manifestations were also considered to determine whether the test result was indicative of the corresponding bacterial infection or just colonization; (2) viral infection, in which positive viral DNA results indicated a viral infection; (3) infection with *Mycoplasma pneumoniae* (MP): (1) serum MP antibody ≥1:32, (2) serum MP antibody ≥1:160 and positive MP PCR test, (3) the MP antibody titer of the recovery phase and acute phase increased or decreased by four times or more; and (4) for fungal infection, clinical diagnosis can be made based on high-risk factors, clinical manifestations suggesting signs of fungal infection, diagnostic imaging features, serum specimens, and a positive (or) BALF G test or GM test (Quan and Tianyou, 2022).

This study used an electronic medical records system to collect medical records, and SPSS 26.0 software was used for data analysis. The measurement data are expressed as the mean ± standard deviation or median (interquartile range). The categorical variables and count data are expressed as the case number and rate. The differences in the count data between the two groups were analyzed using a paired chi-squared test. A *p*-value of <0.05 indicated statistical significance.

## 3 Results

### 3.1 General clinical data of the children

A total of 63 lower respiratory tract samples from 60 children were included: 51 from alveolar lavage fluid, 9 from deep sputum, 2 from pulmonary abscess puncture fluid, and 1 from pleural effusion. Among the 60 children, 42 were boys and 18 were girls aged 0.13–15 years, with a median age of 2.96 (0.95, 7.00) years. The leukocyte count of the children was 1.63–24.74 × 10^9/L, and the median age of the children was 8.63 (6.82, 14.57) × 10^9/L. Patients with abnormal CRP levels accounted for 33.89%, and patients with abnormal PCT levels accounted for 14.89%. Their respiratory manifestations included fever, coughing, wheezing, chest tightness and shortness of breath, and chest pain. The complications included a history of repeated respiratory tract infection, asthma, ventilator use history, and history of major surgery.

### 3.2 mNGS detection results

#### 3.2.1 Pathogen type distribution

**Figure 1:**
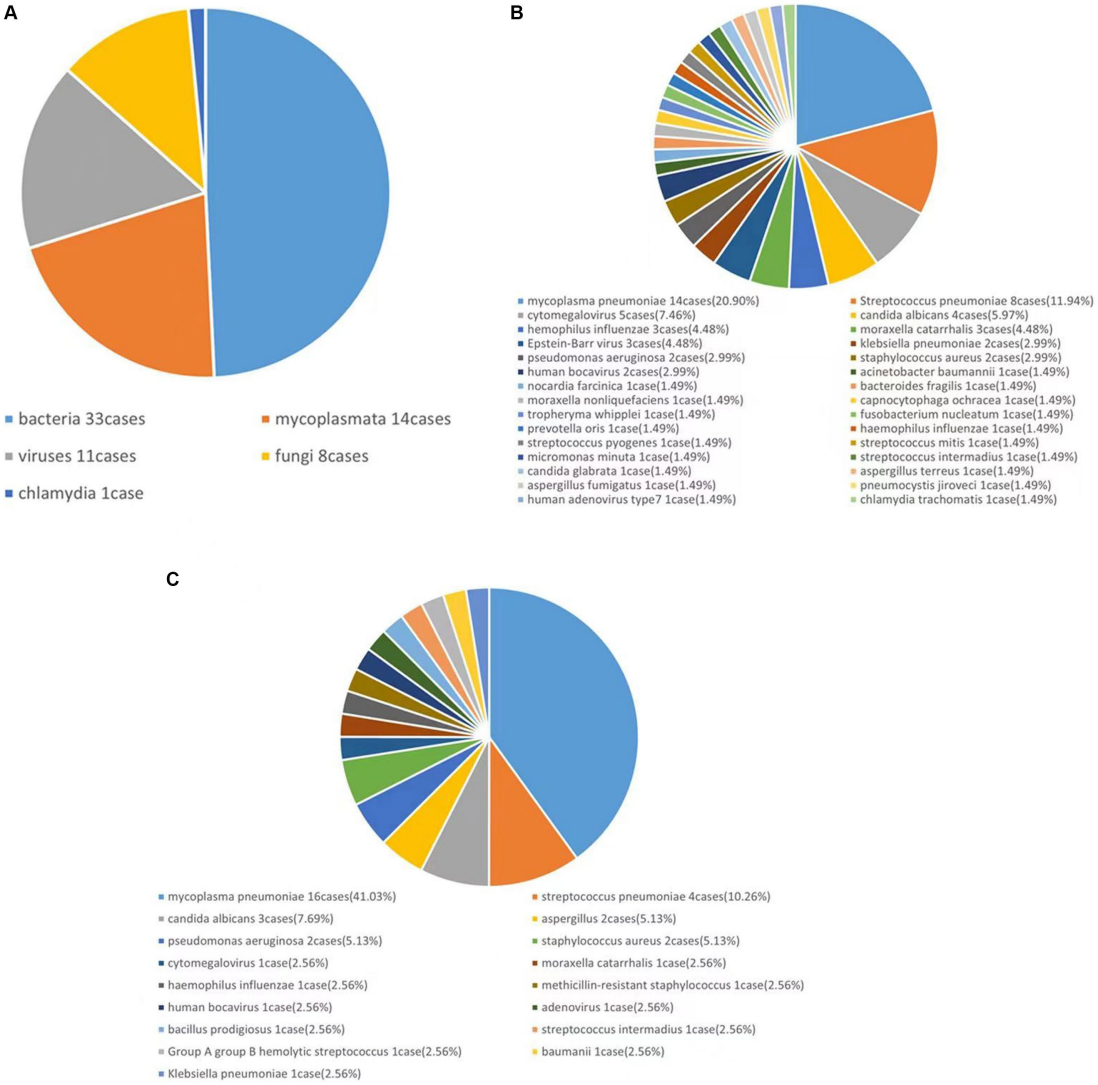
mNGS and traditional method detection results.

A total of 63 lower respiratory tract specimens were collected, 43 of which were positive. In total, five types of bacteria—DNA viruses, fungi, Mycoplasma, and Chlamydia—30 species and 67 strains of microorganisms were collected. Among all the microorganisms, bacteria accounted for 49.25% (33 strains), viruses accounted for 16.42% (11 strains), fungi accounted for 11.94% (8 strains), Mycoplasma accounted for 20.90% (14 strains) and Chlamydia accounted for 1.50% (1 strain), as shown in Figure 1A.

#### 3.2.2 Pathogen distribution

In this study, a total of 30 species and 67 strains of pathogens were detected using mNGS and are listed as follows in descending order of percentage: MP, 20.90% (14 strains); *Streptococcus pneumoniae,* 11.94% (8 strains); cytomegalovirus, 7.46% (5 strains); and *Candida albicans,* 5.97% (4 strains) (Figure 1B).

### 3.3 Results of traditional method detection

The traditional laboratory detection method detected 35 positive pathogens, including bacteria, viruses, fungi, and Mycoplasma. There were 17 species and 39 strains of microorganisms in total, which were ranked from highest to lowest in percentage order as follows: MP 41.03% (16 strains), *Streptococcus pneumoniae* 10.26% (4 strains), *Candida albicans* 7.69% (3 strains), and Aspergillus 5.13% (2 strains) (Figure 1C). In comparison, the bacteria detected by mNGS but not by traditional methods included three strains of *Haemophilus parainfluenzae*, one strain of Bacteroides fancilis, one strain of Moraxella non-liqueficiformis, one strain of Fibrophila flavus, one strain of *Fusobacterium nucleatum*, one strain of Prevotella stoma, one strain of Nocardia meliosum, one strain of *Streptococcus pyogenes*, one strain of Whipple’s trophosis, one strain of Streptococcus light, and one strain of Micromicroomonas microensis. There was one strain of the Epstein–Barr virus, and the fungi included one strain of Candida glabra, one strain of Pneumospora yersoni, and one strain of *Chlamydia trachomatis*. The bacteria detected by traditional methods but not by mNGS included *Serratia marcescens* and Streptococcus B group A.

### 3.4 Distribution of multiple infections

The pathogen infection categories of 60 patients were counted, and the pathogenic microorganisms were categorized according to their clinical data, mNGS, and traditional pathogen detection results. Among them, 21 patients had mixed infections, 9 had triple or more mixed infections, 12 had double mixed infections, 25 had single infections, and another 14 had no clear pathogen. MP combined with other pathogens was the most common mixed infection, and MP was the most common single infection (Table 1).

**Table 1:** Distribution of multiple infections.

	Four or more infections (number of cases)	Triple infection (number of cases)	Double infection (number of cases)	Single infection (number of cases)
Type	*Streptococcus intermedius* + Micromicomonas + *Fusobacterium nucleatum* + sputum + flava (1)	*Mycoplasma pneumoniae* + *Candida albicans* + *Haemophilus parainfluenzae* (1)	*Mycoplasma pneumoniae* + *Candida albicans* (1)	*Mycoplasma pneumoniae* (11)
	Light Streptococcus + EB virus + cytomegalovirus + *Candida albicans* (1)	*Mycoplasma pneumoniae* + *Candida albicans* + Prevotella stomales (1)	*Mycoplasma pneumoniae* + Cytomegalovirus (1)	*Streptococcus pneumoniae* (4)
		*Streptococcus pneumoniae* + human Boca virus + cytomegalovirus (1)	*Mycoplasma pneumoniae* + human Boca virus (1)	*Pseudomonas aeruginosa* (1)
		Cytomegalovirus + Nas. gangrenis + Pneumocystis (1)	*Mycoplasma pneumoniae* + *Bacteroides fragilis* (1)	*Haemophilus parainfluenzae* (1)
		*Candida albicans* + cytomegalovirus + methicillin-resistant *Staphylococcus aureus* (1)	*Mycoplasma pneumoniae* + EB virus (1)	Influenza bloodophilia (1)
		*S. pyogenes* + *Streptococcus pneumoniae* + *M. catarrhalis* (1)	*Acinetobacter baumannii* + *Klebsiella pneumoniae* (1)	*Staphylococcus aureus* (1)
		Streptococcus haemolytica + Mor. catarrhalis + non-liquefied Moraxella (1)	*Acinetobacter baumannii* + parainfluenzae (1)	*K. pneumoniae* (1)
			*Streptococcus pneumoniae* + *Staphylococcus aureus* (1)	*Chlamydia trachomatis* (1)
			*M. catarrhalis* + Whipple Trotrophs (1)	Aspergillus species (2)
			Human Boca virus + *Candida glabrata* (1)	*Aspergillus fumigatus* (1)
			Adenovirus + *Candida albicans* (1)	*Serratia marcescens* (1)
			*Pseudomonas aeruginosa* + *Staphylococcus aureus* (1)	

### 3.5 Comparison of the positive rate between mNGS and traditional methods

The results of all 63 lower respiratory tract specimens from 60 patients were statistically analyzed. The positive rate by mNGS was 90.48%, and the positive rate using the traditional method was 61.90% (*p* = 0.050). The microbial species were divided into five types according to their classification: G+ bacteria, G- bacteria, viruses, fungi, and clade/Chlamydia. The positive rates of the two detection methods for the different types of pathogens were determined. The results are shown in Table 2. The results showed that the rate of positive mNGS results for the detection of G+ bacteria was significantly greater than that of the traditional method (*p* < 0.05). However, there was no significant difference in the percentage of G- bacteria, viruses, fungi, or clade/Chlamydia-positive bacteria.

**Table 2:** Positivity rates and comparisons between mNGS and traditional methods for different types of pathogens.

Type	Positive mNGS rate (%)	Traditional method positive rate (%)	*p*
G+ fungus	22.41	10.34	0.039
G-fungus	18.97	13.80	0.508
Virus	13.79	3.45	0.070
Fungus	15.52	8.62	0.289
Clade/Chlamydia	27.59	29.31	1.000

### 3.6 Comparison of diagnostic performance between mNGS and traditional detection methods

#### 3.6.1 Comparison of the diagnostic efficacy of mNGS and traditional methods in children with refractory pneumonia

The pathogen status was determined according to the clinician’s evaluation combined with the clinical manifestations of the children, relevant examinations, and comprehensive judgment of the two etiological detection methods, and a diagnosis of the pathogen causing the pneumonia was made. The sensitivity, specificity, positive predictive value, negative predictive value, and clinical consistency of mNGS and traditional methods were calculated, with the diagnosed pathogens as the gold standard. The results showed that in children with refractory pneumonia, the sensitivity, specificity, positive predictive value, and negative predictive value of mNGS were 95.45, 37.50, 80.77, and 75.00%, respectively. The values of the traditional method were 72.72, 62.50, 84.21, and 45.45%. The clinical compliance of mNGS was 80.00%, while that of traditional methods was 70.00% (Table 3).

**Table 3:** Diagnostic performance of mNGS and traditional methods in children with refractory pneumonia with confirmed and undiagnosed pathogens.

Divided into groups	Sensitivity (%)	Specificity (%)	Positive predictive value (%)	Negative predictive value (%)	Conformity (%)
mNGS	95.45	37.50	80.77	75.00	80.00
Conventional method	72.72	62.50	84.21	45.45	70.00

#### 3.6.2 Comparison of the diagnostic efficacy of mNGS and traditional methods for different types of pathogens

The diagnostic performance of mNGS and traditional methods for various types of pathogens were analyzed according to the type of confirmed pathogen (Table 4). The sensitivity of mNGS for G+ bacteria was relatively high, and the difference from traditional methods was statistically significant (*p* < 0.05); however, there was no statistically significant difference in the sensitivity of MNGS for G- bacteria, fungi, or Mycoplasma/Chlamydia (*p* > 0.05). Due to the small sample size, statistical analysis could not be conducted on viruses.

**Table 4:** Diagnostic performance of mNGS and traditional methods in children diagnosed with refractory pneumonia versus those without confirmed different types of pathogens.

Type	Pathogen detection method	Sensitivity (%)	Specificity (%)	Positive predictive value (%)	Negative predictive value (%)
G+ fungus	mNGS	92.86	72.73	52.00	96.97
	Conventional method	42.86	93.18	66.67	83.67
G-fungus	mNGS	78.57	72.73	47.83	91.43
	Conventional method	57.14	97.73	88.89	87.76
Virus	mNGS	88.89	83.67	50.00	97.62
	Conventional method	22.22	100.00	100.00	87.50
Fungus	mNGS	81.82	93.62	75.00	95.65
	Conventional method	45.45	89.36	50.00	87.50
Mycoplasma/Chlamydia	mNGS	84.21	100.00	100.00	92.86
	Conventional method	89.47	94.87	89.47	94.87

## 4 Discussion

Pulmonary infection is a common class of respiratory system disease. Using clinical manifestations and imaging findings, it is often difficult to identify the pathogen responsible for lung infection, and the processing of samples, sample quality, and detection time limit the practicality of traditional clinical microbial detection methods (National Institute for Health and Care Excellence, 2014). Incorrect empirical treatment can lead to aggravation and the emergence of drug-resistant bacteria. The emergence of the second generation of mNGS provides a new approach to the diagnosis of microorganisms and, to some extent, overcomes sample and time limitations. Next-generation sequencing technology has been applied to the diagnosis of clinical infectious diseases and has achieved good results, providing new information for accurate diagnosis and treatment.

In our study, after a systematic comparison of mNGS and traditional detection methods, we found that mNGS has several advantages. Specifically, mNGS has a broader pathogen detection spectrum and can quickly identify pathogens, especially under rare or harsh culture conditions. We detected rare bacteria, such as *Aspergillus fumigatus* and Pneumocymonia, by mNGS. mNGS provides clues for the detection of rare pathogens, such as Nocardia, Aspergillus, Leptospira, Plasmodium, and others. It can also help detect new microorganisms, such as novel coronaviruses (Chen et al., 2020), but the detection of pathogenic bacteria is limited by the database, and the gene database needs further improvements. In addition, mNGS can make accurate diagnoses from the perspective of molecular diagnosis and is helpful for the study of airway flora distribution (Li et al., 2020). Due to the lack of unified interpretation standards, it is necessary to carefully interpret the report sheet.

In this study, we retrospectively analyzed the application value of mNGS in the etiological diagnosis of refractory pneumonia in children. A total of 60 children were enrolled in this study. The lower respiratory tract specimens were collected and tested for etiology by mNGS and traditional respiratory methods. The results showed that mNGS detected a wide variety of microorganisms, including fastidious bacteria and rare bacteria that were not detected by traditional methods. In this study, mNGS detected 30 species and 67 strains of microorganisms, among which bacteria accounted for 49.25% and Gram-negative bacteria accounted for 25.37%. From high to low prevalence, *Haemophilus parainfluenzae*, *Moraxella catarrhalis*, *Klebsiella pneumoniae,* and *Pseudomonas aeruginosa* accounted for 23.88%, and *Streptococcus pneumoniae* accounted for the greatest proportion. The pathogens detected by mNGS from most to least were MP (20.90%, 14 strains), *Streptococcus pneumoniae* (11.94%, 8 strains), cytomegalovirus (7.46%, 5 strains), and *Candida albicans* (5.97%, 4 strains). Some rare microorganisms, such as *Aspergillus fumigatus*, Pneumocystis jasinii, and Nocardia melis, were also detected. Many studies have shown the advantages of mNGS in the diagnosis of bacterial infections, especially with fastidiosa, rare bacteria, and atypical pathogenic bacteria (Burnham et al., 2014; Wilson et al., 2014; Brown et al., 2015; Naccache et al., 2015; Simner et al., 2018; Chiu and Miller, 2019).

This study showed that bacteria accounted for the largest proportion of infectious pathogens in children with refractory pneumonia and accounted for a high proportion of the species identified by both mNGS and traditional methods. However, mNGS detected more bacteria that were not detected by traditional methods, such as Nocardia meliogii, Whipple’s trophozoa, Fibrophilia fusarium, and *Streptococcus pyogenes*. Due to the limitations of traditional pathogen detection methods, it takes a long time for these detection methods to identify some pathogens that are not easy to detect, such as Nocardia and fungi; therefore, the pathogen cannot be detected in time (Miao et al., 2018; Zhou et al., 2019). Nocardia is a rare infectious disease with a high mortality rate. Although Nocardia can be grown on blood agar or sediment culture media, the culture of this organism is slow, taking 2 days to several weeks. The detection rate of Nocardia in the clinic is not high. Whipple’s trophophilia and fusotrophilia CO2 also have high culture requirements, and Whipple’s trophophilia cannot be detected by conventional blood and tissue culture. PCR plays a certain role in the detection of Whipple’s trophophilia, but its yield depends on the influence of specimen type. CO_2_ fibrophilia are facultative anaerobes or microaerobes. These species require CO_2_ for growth, and they have high nutritional and incubation requirements. These fungi require a long period of culture to grow, and they are difficult to detect by traditional methods (Qin et al., 2020). Therefore, traditional methods for the detection of these strains are limited.

In addition, the results of this study showed that there was no significant difference in the percentage of positive fungal or viral results between mNGS and traditional culture. This finding is different from other research results (Deng et al., 2022). On the one hand, the fungi of Firmicutes have a cell wall that is difficult to destroy during mNGS detection, resulting in the limited release of nucleic acid, so the detection rate of intracellular bacteria and Firmicutes by mNGS is low (Han and Liu, 2022). On the other hand, the sample size of this study was small, resulting in bias in the results. The rate of virus detection by mNGS and traditional methods was lower, in disagreement with other studies (Goldberg et al., 2015; Niu et al., 2020). This may be related to the fact that only DNA sequence results, not RNA sequence results, were included in this study. Since RNA viruses have easily degraded nucleic acids that require strict transportation and preservation conditions, mNGS detection of them is still difficult. RNA viruses, such as respiratory syncytial virus and influenza virus, do account for a considerable proportion of respiratory infectious diseases in children (Author, 2019).

The children in this study were relatively young, had weak resistance, and had immature bronchopulmonary development. The risk of pulmonary coinfection in these patients is greater than that in adults, especially in immunocompromised children, such as in children with hematological malignancies and long-term use of glucocorticoids. In this study, a significant proportion of children with refractory pneumonia had mixed infections, which was consistent with other findings (Chen, 2021; Guo et al., 2021). There were 21 cases of mixed infection, 9 cases of three or more mixed infections, 12 cases of double mixed infection, and 22 cases of single infection. Among them, mixed infection with MP was most common in combination with other pathogens, such as MP combined with *Candida albicans*, MP Boca virus, and the most common single infection with MP. In this study, compared to the detection of pathogens in the two groups of children, the mNGS results showed mostly mixed infections, a result of the sequencing advantage of mNGS. mNGS can detect rare microbial information that cannot be detected by more traditional tests, and the etiology diagnosis rate of mixed infections in children is relatively high. In addition, in 14 children in this study, the pathogen was not identified, which could be because only DNA virus information, not RNA virus information, was available.

In addition, our results suggest that mNGS has better diagnostic performance than traditional culture methods. In children with refractory pneumonia, the sensitivity and negative predictive value of mNGS for detecting G+ bacteria are greater, suggesting that negative mNGS results have greater significance in excluding diagnoses. In treatment, the high sensitivity and negative predictive value of mNGS can be used to eliminate the original suspected pathogenic bacteria, stop the original treatment plan, and reduce the unnecessary use of antibiotics. The above findings are in line with Wang et al. (2019), and the clinical consistency of the diagnosis of mNGS is greater than that of traditional methods. However, there was no statistically significant difference in G- bacteria, fungi, or Mycoplasma/Chlamydia in this study, which may be related to the small sample size.

In conclusion, compared to traditional methods, mNGS has obvious advantages in diagnosing and guiding the treatment of children with refractory pneumonia, especially in terms of bacteria, rare microorganisms, and mixed infections. However, mNGS also has some shortcomings. First, mNGS can detect all microorganisms in the sample, which makes it impossible to distinguish between pathogenic bacteria and colonizing bacteria in specimens from bacterial environments such as the respiratory tract, making the interpretation of results subjective to a certain extent. 2. At present, the price and cost of mNGS detection are still significantly greater than those of unified detection methods, which limits its clinical application. Therefore, mNGS is more suitable for difficult and critically ill patients or for use in combination with traditional etiological tests.

Our study has some limitations. First, as a retrospective study, it has inevitable selection bias and recall bias. Second, the lack of RNA sequencing leads to a better assessment of the diagnostic value of traditional methods and mNGS, especially for viral infections. Third, the small sample size of this study, especially for fungal pneumonia, could bias the results. Finally, the interpretation of pathogens detected by mNGS depends more on the subjective judgment of clinicians than on diagnostic confirmation because there is no unified standard for unbiased detection of mNGS, and it is difficult to distinguish between colonizing and infectious pathogens.

## 5 Conclusion

The efficacy of mNGS in the etiological diagnosis of refractory pneumonia in children is better than that of traditional methods. The application of mNGS can significantly improve the detection rate of pathogens in children with refractory pneumonia. The sensitivity and the negative predictive value of mNGS detection are greater, allowing for the identification of pathogenic microorganisms as soon as possible, elimination of the original suspected pathogenic bacteria, improvement of the cure rate, and reduction in the unnecessary use of antibiotics.

## Data availability statement

The raw data supporting the conclusions of this article will be made available by the authors, without undue reservation.

## Ethics statement

The studies involving humans were approved by Ethics Committee of Children’s Hospital Affiliated to the Capital Institute of Pediatrics. The studies were conducted in accordance with the local legislation and institutional requirements. Written informed consent for participation was not required from the participants or the participants’ legal guardians/next of kin because this study was a retrospective study.

## Author contributions

Y-nW: Investigation, Methodology, Writing – original draft, Conceptualization, Data curation, Software, Validation, Visualization. Y-tW: Data curation, Resources, Writing – original draft, Investigation, Validation, Visualization. LC: Funding acquisition, Methodology, Project administration, Resources, Supervision, Writing – review & editing. W-qN: Writing – review & editing, Formal analysis, Resources.

## Footnotes

^1^Downloaded from: https://www.ncbi.nlm.nih.gov/.

## References

[ref1] Expert consensus group for applications of metagenomic analysis and diagnostic techniques in acute and critical infections (2019). Application of metagenomic analysis and diagnostic techniques to the expert consensus group for critically ill infections. Expert consensus on the application of metagenomic analysis and diagnostic techniques in acute and severe infections. Chinese J. Emerg. Med. 2, 151–155. doi: 10.3760/cma.j.issn.1671-0282.2019.02.005 (in Chinese).

[ref2] Brown J. R. Morfopoulou S. Hubb J. Emmett W. A. Ip W. Shah D.. (2015). Astrovirus VA1/HMO-C: an increasingly recognized neurotropic pathogen in immunocompromised patients. Clin. Infect. Dis. 60, 881–888. doi: 10.1093/cid/ciu940, PMID: 25572899 PMC4345817

[ref3] Burnham J. P. Thomas B. S. Trevino S. E. McElvania Tekippe E. Burnham C. A. Kuhlmann F. M. (2014). De novo meningitis caused by *Propionibacterium acnes* in a patient with metastatic melanoma. J. Clin. Microbiol. 52, 1290–1293. doi: 10.1128/JCM.02755-13, PMID: 24478417 PMC3993499

[ref4] Chen W. Y. (2021). Clinical characteristics of 295 children with refractory pneumonia. Dali: Dali University (in Chinese).

[ref5] Chen L. Liu W. Zhang Q. Xu K. Ye G. Wu W.. (2020). RNA based mNGS approach identifies a novel human coronavirus from two individual pneumonia cases in 2019 Wuhan outbreak. Emerg Microbes Infect 9, 313–319. doi: 10.1080/22221751.2020.1725399, PMID: 32020836 PMC7033720

[ref6] Chinese Thoracic Society (2023). Consensus of clinical pathways of metagenomic next-generation sequencing test in diagnosis of lower respiratory tract infections in China. Chinese Journal of Tuberculosis and Respiratory Diseases 04, 322–335. doi: 10.3760/cma.j.cn112147-20220701-00553 (in Chinese). 36787941

[ref7] Chiu C. Y. Miller S. A. (2019). Clinical metagenomics. Nat. Rev. Genet. 20, 341–355. doi: 10.1038/s41576-019-0113-7, PMID: 30918369 PMC6858796

[ref8] Darby J. B. Singh A. Quinonez R. (2017). Management of Complicated Pneumonia in childhood: a review of recent literature. Rev. Recent Clin. Trials 12, 253–259. doi: 10.2174/1574887112666170816144110, PMID: 28814258

[ref9] Deng W. Xu H. Wu Y. Li J. (2022). Diagnostic value of bronchoalveolar lavage fluid metagenomic next-generation sequencing in pediatric pneumonia. Front. Cell. Infect. Microbiol. 12:950531. doi: 10.3389/fcimb.2022.950531, PMID: 36389175 PMC9648200

[ref10] GBD 2019 Under-5 Mortality Collaborators (2021). Global, regional, and national progress towards sustainable development goal 3.2 for neonatal and child health: all-cause and cause-specific mortality findings from the global burden of disease study 2019. Lancet 398, 870–905. doi: 10.1016/S0140-6736(21)01207-1 34416195 PMC8429803

[ref11] Goldberg B. Sichtig H. Geyer C. Ledeboer N. Weinstock G. M. (2015). Making the leap from research laboratory to clinic: challenges and opportunities for next-generation sequencing in infectious disease diagnostics. MBio 6, e1815–e1888. doi: 10.1128/mBio.01888-15 PMC4669390 26646014

[ref12] Guo P. B. Wang Y. H. Zhang B. Zhang X. M. (2021). Analysis of pathogen examination results of 11299 children with acute respiratory tract infection in Zhengzhou area. J. Clin. Pulmonol. 26, 174–177. doi: 10.3969/j.issn.1009-6663.2021.02.003 (in Chinese).

[ref13] Han S. Y. Liu J. H. (2022). Clinical application value of metagenomic next generation sequencing in difficult infectious diseases. Chin J Contemp Pediatrics 24, 210–215. doi: 10.7499/j.issn.1008-8830.2110064 (in Chinese). PMC8884048 35209988

[ref14] He C. Liu L. Chu Y. Perin J. Dai L. Li X.. (2017). National and subnational all-cause and cause-specific child mortality in China, 1996-2015: a systematic analysis with implications for the sustainable development goals. Lancet Glob. Health 5, e186–e197. doi: 10.1016/S2214-109X(16)30334-5, PMID: 28007477 PMC5250590

[ref15] Li Y. Sun B. Tang X. Liu Y. L. He H. Y. Li X. Y.. (2020). Application of metagenomic next-generation sequencing for bronchoalveolar lavage diagnostics in critically ill patients. Eur. J. Clin. Microbiol. Infect. Dis. 39, 369–374. doi: 10.1007/s10096-019-03734-5, PMID: 31813078 PMC7102353

[ref16] Miao Q. Ma Y. Wang Q. Pan J. Zhang Y. Jin W.. (2018). Microbiological diagnostic performance of metagenomic next-generation sequencing when applied to clinical practice. Clin. Infect. Dis. 67, S231–S240. doi: 10.1093/cid/ciy693, PMID: 30423048

[ref17] Naccache S. N. Peggs K. S. Mattes F. M. Phadke R. Garson J. A. Grant P.. (2015). Diagnosis of neuroinvasive astrovirus infection in an immunocompromised adult with encephalitis by unbiased next-generation sequencing. Clin. Infect. Dis. 60, 919–923. doi: 10.1093/cid/ciu912, PMID: 25572898 PMC4345816

[ref18] National Institute for Health and Care Excellence (2014). Pneumonia: Diagnosis and Management of Community- and Hospital-Acquired Pneumonia in adults. London: National Institute for Health and Care Excellence. 25520986

[ref19] Niu Y. Y. Wu X. H. Ying K. J. (2020). The advantage of bronchoalveolar lavage fluid macrogene next generation sequencing for pathogen detection of lower respiratory tract infections. Chinese J. Pract. Internal Med. 40, 754–758. doi: 10.19538/j.nk2020090111 (in Chinese).

[ref20] Qin W. C. Sun G. Y. Gao Y. Mou J. Fang Y. H. (2020). A case report of endophthalmitis caused by sputum producing fibrophilia carbon dioxide infection. Int J Lab Med 41, 2303–2304. doi: 10.3969/j.issn.1673-4130.2020.18.032 (in Chinese).

[ref21] Quan L. Tianyou W. (2022). Expert consensus on clinical practice of invasive pulmonary fungal infections in children (2022). Zhonghua Er Ke Za Zhi 60, 274–282. doi: 10.3760/cma.j.cn112140-20220210-00112 35385930

[ref22] Simner P. J. Miller S. Carroll K. C. (2018). Understanding the promises and hurdles of metagenomic next-generation sequencing as a diagnostic tool for infectious diseases. Clin. Infect. Dis. 66, 778–788. doi: 10.1093/cid/cix881, PMID: 29040428 PMC7108102

[ref23] Smalley N. MacLaren G. Best D. Paul E. Butt W. (2012). Outcomes in children with refractory pneumonia supported with extracorporeal membrane oxygenation. Intensive Care Med. 38, 1001–1007. doi: 10.1007/s00134-012-2581-5, PMID: 22543425

[ref24] Torres A. Chalmers J. D. Dela C. C. Dominedò C. Kollef M. Martin-Loeches I.. (2019). Challenges in severe community-acquired pneumonia: a point-of-view review. Intensive Care Med. 45, 159–171. doi: 10.1007/s00134-019-05519-y, PMID: 30706119 PMC7094947

[ref25] Wang J. Han Y. Feng J. (2019). Metagenomic next-generation sequencing for mixed pulmonary infection diagnosis. BMC Pulm. Med. 19:252. doi: 10.1186/s12890-019-1022-4, PMID: 31856779 PMC6921575

[ref26] Wilson M. R. Naccache S. N. Samayoa E. Biagtan M. Bashir H. Yu G.. (2014). Actionable diagnosis of neuroleptospirosis by next-generation sequencing. N. Engl. J. Med. 370, 2408–2417. doi: 10.1056/NEJMoa1401268, PMID: 24896819 PMC4134948

[ref27] Zhou X. Wu H. Ruan Q. Jiang N. Chen X. Shen Y.. (2019). Clinical evaluation of diagnosis efficacy of active *Mycobacterium tuberculosis* complex infection via metagenomic next-generation sequencing of direct clinical samples. Front. Cell. Infect. Microbiol. 9:351. doi: 10.3389/fcimb.2019.00351, PMID: 31681628 PMC6813183

